# Adiponectin Associates with Rheumatoid Arthritis Risk in Overweight and Obesity Independently of Other Adipokines

**DOI:** 10.3390/jcm10132791

**Published:** 2021-06-25

**Authors:** Yuan Zhang, Linda Johansson, Johanna Andersson-Assarsson, Magdalena Taube, Markku Peltonen, Per-Arne Svensson, Christian Herder, Anna Rudin, Lena Carlsson, Solbritt Rantapää-Dahlqvist, Cristina Maglio

**Affiliations:** 1 Department of Rheumatology and Inflammation Research, Sahlgrenska Academy, University of Gothenburg, SE-40530 Gothenburg, Sweden; yuan.zhang@gu.se (Y.Z.); Anna.Rudin@microbio.gu.se (A.R.); 2 Wallenberg Center for Molecular and Translational Medicine, University of Gothenburg, SE-40530 Gothenburg, Sweden; 3 Public Health and Clinical Medicine/Rheumatology, Umeå University, SE-90587 Umeå, Sweden; linda.e.johansson@umu.se (L.J.); solbritt.rantapaa.dahlqvist@umu.se (S.R.-D.); 4 Department of Molecular and Clinical Medicine, Sahlgrenska Academy, University of Gothenburg, SE-41345 Gothenburg, Sweden; johanna.andersson@medic.gu.se (J.A.-A.); magdalena.taube@wlab.gu.se (M.T.); per-arne.svensson@medic.gu.se (P.-A.S.); lena.carlsson@medic.gu.se (L.C.); 5 Public Health Promotion Unit, National Institute for Health and Welfare, FI-00271 Helsinki, Finland; markku.peltonen@thl.fi; 6 Department of Neurobiology, Care Sciences and Society, Karolinska Institutet, SE-17164 Solna, Sweden; 7 Institute of Health and Care Sciences, Sahlgrenska Academy, University of Gothenburg, SE-41346 Gothenburg, Sweden; 8 Institute for Clinical Diabetology, German Diabetes Center, Leibniz Center for Diabetes Research, Heinrich-Heine University Duesseldorf, 40225 Duesseldorf, Germany; christian.herder@ddz.de; 9 German Center for Diabetes Research (DZD), Partner Düsseldorf, 85764 Neuherberg, Germany; 10 Department of Endocrinology and Diabetology, Medical Faculty and University Hospital Düsseldorf, Heinrich-Heine-University Düsseldorf, 40204 Düsseldorf, Germany; 11 Rheumatology Clinic, Sahlgrenska University Hospital, Region Västra Götaland, SE-41345 Gothenburg, Sweden

**Keywords:** adiponectin, adipokines, rheumatology, obesity, overweight, case control study

## Abstract

We recently reported that increased serum adiponectin was associated with rheumatoid arthritis (RA) risk in subjects with obesity. We hereby aim to determine if other adipokines associate with RA risk and if the association between adiponectin and RA is independent of other adipokines. Two nested-case control studies were performed in two different cohorts: 82 participants of the Swedish Obese Subjects (SOS) study who developed RA during follow-up matched with 410 controls, and 88 matched pairs from the Medical Biobank of Northern Sweden. Baseline levels of circulating adipokines were measured using ELISA. In a multivariable analysis in the SOS cohort, higher adiponectin was associated with an increased risk of RA independently of other adipokines (OR for RA risk: 1.06, 95% CI: 1.01–1.12, *p* = 0.02). No association between leptin, resistin, and visfatin levels and the risk of RA was detected. In the cohort from the Medical Biobank of Northern Sweden, higher adiponectin was associated with an increased risk of RA only in participants with overweight/obesity (OR: 1.17, 95% CI: 1.01−1.36, *p* = 0.03), independently of other adipokines. Our results show that in individuals with overweight/obesity, higher circulating levels of adiponectin, but not leptin, resistin, or visfatin, were associated with an increased RA risk.

## 1. Introduction

Adipose tissue is the largest endocrine organ in the human body, which produces large amounts of cell-signaling proteins, called adipokines [1]. Adipokines, such as adiponectin, leptin, resistin, and visfatin, are important regulators of metabolism [2]. In obesity, the balance between pro- and anti-inflammatory adipokines shifts to create a pro-inflammatory environment in the adipose tissue contributing to the chronic low-grade inflammation and the metabolic disorders that characterize obesity [2]. However, obesity is not only a risk factor for metabolic disturbances such as type 2 diabetes or metabolic syndrome but also for inflammatory diseases such as gout, psoriasis, and rheumatoid arthritis (RA) [3,4,5,6,7]. Adipokines, by virtue of their regulatory role in the integration between metabolism and systemic inflammation, are candidates to be involved in the pathogenesis of inflammatory diseases [8,9]. 

Several adipokines have been found elevated in blood from patients with RA, a systemic inflammatory disease affecting mainly the joints [10]. However, it is unknown whether they contribute to the development of the disease or if they are unspecific markers of inflammation [9]. An interesting example is adiponectin, which is the most abundant adipokine circulating in blood [11]. As opposed to other adipokines, circulating adiponectin levels are low in patients with obesity, type 2 diabetes, and metabolic syndrome [12,13]. On the other hand, circulating adiponectin levels are elevated in inflammatory conditions such as RA, and synovial fluids from patients with RA show higher concentrations of adiponectin compared to those from patients with osteoarthritis [9,14]. 

Other adipokines, such as leptin, resistin, and visfatin, have been associated with RA. Amongst those adipokines, leptin is the one with the most robust association with RA, as high circulating leptin levels correlate with RA disease activity and leptin is also able to stimulate RA fibroblast-like synoviocytes to produce pro-inflammatory cytokines [15,16]. Circulating resistin levels have also been associated with RA, although with inconsistent results [17,18]. However, despite some controversial results, it is generally agreed that resistin is involved in the pathogenesis of RA. Intra-articular injection with resistin causes joint inflammation similar to RA in a murine model, and recombinant resistin is able to induce the production of pro-inflammatory factors from human blood mononuclear cells and fibroblast-like synoviocytes from patients with RA [19,20]. Visfatin is an adipokine mainly produced by visceral adipose tissue and by neutrophils [8]. Patients with RA have elevated levels of visfatin in blood and in inflamed synovial tissue compared to controls, and visfatin is shown to trigger the motility and cytokine production in fibroblast-like synoviocytes [21,22].

Blood samples from patients who later developed RA show signs of inflammation several years before the disease onset. Autoantibodies, cytokines, and biomarkers of inflammation can be elevated up to 10 years before the first signs of RA [23,24]. We have recently shown that serum adiponectin at baseline associated with the future development of RA independently of other risk factors in the Swedish Obese Subjects (SOS) study, a large cohort of patients with obesity followed up for up to 29 years [25].

The objective of the current study was to determine if circulating adipokines other than adiponectin also associate with the future development of RA and if the association between circulating levels of adiponectin and higher risk of RA is independent of other adipokines. To answer these questions, we performed two nested-case control studies, one within the SOS study, which includes only patients with obesity, and the other one at a general population level within the Medical Biobank of Northern Sweden.

## 2. Results

### 2.1. Baseline Characteristics of the Nested Case-Control Cohort from the SOS Study

Baseline characteristics of the 82 cases before the development of RA and the 410 matched controls are shown in Table 1. As a result of the matching procedure, the two groups had similar characteristics regarding age, the proportion of men/women, body mass index (BMI), smokers/non-smokers, and patients who underwent bariatric surgery or received conventional treatment for obesity. Cases had higher levels of C-reactive protein (CRP) and erythrocyte sedimentation rate (ESR) than the matched controls. No significant difference was observed in the serum levels of adipokines between the two groups. We performed a Spearman’s correlation test, which showed a poor or mild correlation among adipokine levels (Spearman’s coefficients < 0.5 for all the combinations).

### 2.2. Baseline Characteristics of the Nested Case-Control Cohort from the Medical Biobank of Northern Sweden

Table 2 shows baseline characteristics of the cohort from the Medical Biobank of Northern Sweden, both cases and matched controls. The two groups had similar characteristics including age, the proportion of men/women, and BMI. Cases were more frequently smokers than the participants from the control group. No difference in the plasma levels of adipokines was detected between the two groups. Circulating levels of adipokines showed poor correlation (Spearman’s coefficients < 0.1 for all the combinations).

### 2.3. Multivariable Analyses for the Incidence of RA in the Nested Case-Control Cohort from the SOS Study

Table 3 shows the multivariable conditional logistic regression analyses for the risk of RA in the SOS nested case-control cohort. When only adipokines are included in the model, solely the association between adiponectin levels and risk of RA was close to significance (OR: 1.05, per 1000 ng/mL; 95% CI: 1.00–1.10, *p* = 0.05; Table 3 (A Model 1)). The analysis was not adjusted for age, sex, BMI, or smoking as those variables were used for matching, and they were not different between the two groups (Table 1). As CRP and ESR levels were different between cases and controls (Table 1), we included those two variables in the analysis. As shown in Table 3 (B Model 2), increased serum levels of adiponectin were associated with a higher risk of RA (OR: 1.06; per 1000 ng/mL, 95% CI: 1.01–1.12, *p* = 0.02) independently of other adipokines as well as inflammation markers CRP and ESR. As previously shown in the same cohort [25,26], CRP was also independently associated with a higher risk of RA (OR: 1.67; per 10 mg/L, 95% CI: 1.17–2.42, *p* = 0.01). ESR and serum levels of leptin, resistin, and visfatin did not show any significant association with the risk of RA.

### 2.4. Multivariable Analysis for the Incidence of RA in the Nested Case-Control Cohort from the Medical Biobank of Northern Sweden

In the cohort from the Medical Biobank of Northern Sweden, no association between adipokines and risk of RA was detected in a multivariable analysis, including plasma adipokine levels and smoking (Table 4A). Smoking was included in the model as the percentage of current or previous smokers was different at baseline between cases and controls (Table 2). CRP and ESR levels were not available at baseline in the cohort from the Medical Biobank of Northern Sweden and therefore could not be included in the analysis. To mimic the conditions of the SOS cohort characterized by subjects affected by obesity, we then stratified the population according to BMI. Plasma levels of adiponectin were associated with a risk of RA (OR: 1.17, 95% CI: 1.01−1.36, *p* = 0.03) independently of other factors in the subgroup having BMI > 25 kg/m^2^ (*n* = 109) but not in the one having BMI ≤ 25 kg/m^2^ (*n* = 67), as shown in Table 4B,C. These analyses were also adjusted for age, sex, and smoking as cases and controls were not matched for these variables when stratifying for BMI. However, similar results were obtained in the unadjusted analysis (data not shown). The interaction between adiponectin levels and BMI on the risk of RA was not significant (*p* = 0.13). Baseline clinical characteristics of the cohort from the Medical Biobank of Northern Sweden after stratification by BMI are shown in Table 5.

## 3. Discussion

By performing two nested case-control studies in cohorts where blood samples and clinical information were available before the diagnosis of RA, we have observed no association between circulating levels of leptin, resistin, and visfatin and the future risk of RA. Only elevated circulating adiponectin levels were associated with an increased risk of developing RA in participants with overweight/obesity independently of other adipokines.

This study confirms what we have shown in a recent publication where increased serum adiponectin levels were associated with the future incidence of RA in participants of the SOS, a longitudinal study including about 4000 subjects with obesity [25]. This association was independent of confounding factors, including bariatric surgery. As circulating levels of other adipokines are known to be elevated in patients with RA compared to controls, we wanted to determine if leptin, resistin, and visfatin levels were also associated with the future development of RA [9]. By performing a nested case-control study within the SOS study based on the same patients with incident RA as in our previous study matched 1:5 with controls without incident RA [25], we did not observe any association between leptin, resistin, and visfatin and the risk of future RA. As expected, serum adiponectin levels were positively associated with an increased risk of developing RA, and this association was independent of other adipokines. As the SOS study only included participants with obesity, we aimed to test our hypothesis at the general population level and therefore measured plasma levels of adiponectin, leptin, and resistin in 88 pre-symptomatic patients before symptom onset of RA and 88 matched controls from the Medical Biobank of Northern Sweden. It was not possible to measure circulating visfatin due to the low amount of available plasma. In this cohort, no adipokine was associated with the development of RA. To mirror the conditions of the SOS study where all the participants are affected by obesity, we decided to stratify the population according to BMI and found that elevated circulating adiponectin levels were associated with an increased risk of RA independently of leptin and resistin only in participants with overweight/obesity. No association of adiponectin, nor other adipokines, and risk of RA was observed in normal-weight participants.

It is unclear why circulating adiponectin was associated with the future risk of RA exclusively in participants with overweight/obesity. A possible explanation is that a chronic pro-inflammatory state as seen in obesity is needed to expose the association between adiponectin levels and RA. Furthermore, as high BMI associates with low circulating adiponectin levels and at the same time with an increased risk of developing RA, it is possible to hypothesize that overweight and obesity uncover the link between adiponectin and risk of RA [12,27]. According to this hypothesis, the association between increased adiponectin levels and the risk of developing future RA is also present at a general population level, but it becomes clearly detectable in subjects with overweight/obesity as they have constitutionally lower circulating levels of adiponectin. However, it is important to point out that this remains a pure hypothesis that we cannot confirm or reject in the present report, as the cohort of the Medical Biobank of Northern Sweden included in this study is not large enough. Moreover, the interaction between adiponectin levels and BMI on the risk of RA was not significant in the same cohort.

The finding that patients with RA have elevated circulating levels of adiponectin compared to controls is known for many years [9,14]. However, as adiponectin has both anti- and pro-inflammatory properties, the increase in adiponectin levels in blood has been hypothesized to be a protective mechanism to counterbalance systemic and local inflammation [28,29]. On the other hand, adiponectin might play an active role in the development of RA as it is able to induce pro-inflammatory responses in cells involved in the pathogenesis of RA, such as fibroblast-like synoviocytes and antibodies against human adiponectin ameliorated rheumatic symptoms in a collagen-induced arthritis mouse model [30,31]. Our previous and current data show that adiponectin levels are associated with the risk of future development of RA in patients with overweight/obesity, thus supporting the hypothesis that this adipokine might play a role in the pathogenesis of RA at least in this group of patients. However, other factors, such as CRP, are known to increase in the blood several years before the onset of RA, and they do not necessarily play a role in the disease development but are unspecific markers of inflammation. Further in vitro studies are needed to determine if adiponectin has a pathogenic role in RA or is a marker of inflammation in the context of RA.

Circulating levels of leptin, resistin, and visfatin have been previously shown to be elevated in patients with RA compared to controls [9,15,17]. Moreover, in vitro studies have demonstrated that leptin, resistin, and visfatin are able to stimulate the production of pro-inflammatory factors in cells from patients with RA [16,19,22]. However, we have recently reported that, in patients with untreated newly diagnosed RA, leptin and resistin are not associated with markers of disease activity nor pro-inflammatory chemokines [32]. Our present study could not detect any association between circulating levels of leptin, resistin, and visfatin and future risk of RA. Taken together, these results might suggest that those three adipokines are neither involved in the initiation of RA nor in the early pre-clinical phases of RA development. However, future studies in a larger cohort are needed to determine if circulating levels of leptin, resistin, and visfatin are associated with the future risk of RA.

Our study has some limitations. We performed two nested case-control studies from two larger cohorts, and the low number of participants included might have affected the results. Specifically, the nested case-cohort study performed within the Medical Biobank of Northern Sweden is rather small. Based on the obtained ORs, this population does not have enough statistical power to allow detecting an association between circulating adipokines on the risk of developing RA at a general population level. Moreover, the two cohorts are very different in terms of baseline characteristics as well as the proportion of cases/controls. The limited number of participants with obesity in the Medical Biobank of Northern Sweden (10 RA cases and 8 matched controls having BMI > 30) did not allow us to stratify this cohort based on BMI equal to 30, and therefore we decided to use BMI equal to 25 (defining overweight) as a cut-off instead. Another limitation of the study is that it was not possible to measure visfatin in the cohort from the Medical Biobank of Northern Sweden due to the lack of plasma samples. Further studies in larger cohorts are warranted to determine if circulating levels of adipokines are able to predict the future development of RA at a general population level.

## 4. Materials and Methods

### 4.1. SOS Study

A nested case-control study was performed within the SOS study. Details about the SOS study, such as recruitment, inclusion criteria, and sample collection and storage, have been previously described [33,34]. Briefly, the ongoing SOS study is a longitudinal non-randomized controlled intervention study investigating the impact of bariatric surgery on mortality and morbidity in patients affected by obesity. Between 1987 and 2001, 4047 individuals with obesity were enrolled in Sweden. Two-thousand and ten patients voluntarily chose bariatric surgery and underwent vertical banded gastroplasty (68%), gastric banding (19%), or gastric bypass (13%). A matched control group including 2037 patients with obesity was created based on 18 different matching variables as previously described [34]. Participants from the control group received conventional treatment for obesity which was provided at their primary health care centers [34,35]. The study was approved by seven regional ethics review boards in Sweden and is registered at https://clinicaltrials.gov/ (accessed on 22 June 2021) with the identifier NCT0147952. All patients gave informed consent to participate in the study.

Diagnoses of RA were retrieved through the Swedish National Patient Register by looking for the following International Classification of Diseases (ICD) codes: 712.38, 712.39 (ICD-8), and 714.0-2 (ICD-9) and M05, M06.0, M06.8, and M06.9 (ICD-10) [26]. SOS study participants were followed up until diagnosis of RA, death, migration, or end of follow-up, which was 31 December 2016. Information on death or migration was obtained from the Cause of Death Register and the Register of the Total Population [36].

**Figure 1:**
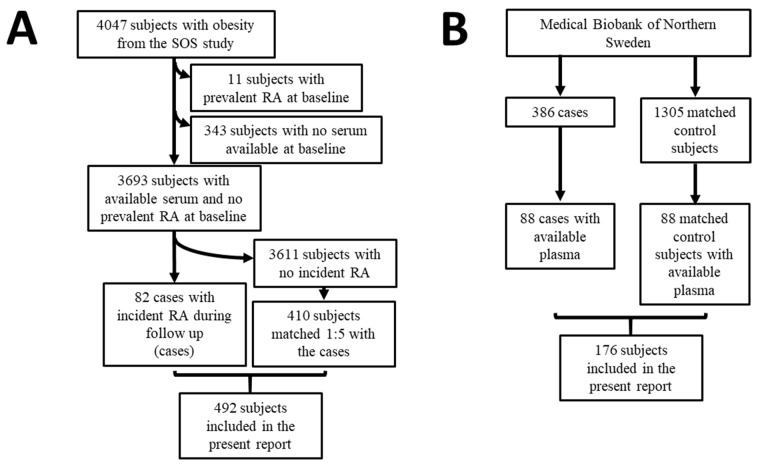
Flow scheme for the present report. (**A**) Nested case-control study from the Swedish Obese Subjects study. (**B**) Nested case-control study within the cohort of the Medical Biobank of Northern Sweden.

Eleven patients had prevalent RA, and 343 had no available serum samples at baseline and were therefore excluded from the analyses (Figure 1A). Among the 3693 participants with available serum and no prevalent RA at baseline, 82 patients developed RA during follow-up (cases). The 82 cases were matched 1:5 with 410 subjects who did not develop RA during follow-up (controls), as depicted in Figure 1A. Matching was performed using the propensity score method based on baseline age, sex, BMI, bariatric surgery yes/no, year of inclusion, and smoking [37]. The median follow-up time was 21 years, ranging from 0 to 29 years in the entire cohort, whereas the median pre-dating time before the diagnosis of RA was 14 years, ranging from 1 to 27 years.

### 4.2. The Cohort from the Medical Biobank of Northern Sweden

A nested case-control study was performed in a group of individuals included in population surveys within the cohort from the Medical Biobank of Northern Sweden. Details about the recruitment, inclusion criteria, and sample collection and storage of the original study have been previously described [24]. Briefly, the register from the Medical Biobank of Northern Sweden was cross-checked with the registers of patients with RA attending the Department of Rheumatology, University Hospital of Umeå, to identify those individuals who had donated blood samples before the symptoms of RA. Diagnosis of RA was based on the 1987 American Rheumatism Association classification criteria [38]. Three-hundred and eighty-six individuals (71 men and 315 women) donating a total of 717 plasma samples were included in the original study (Figure 1B). A total of 1305 controls, matched for age, sex, and date of blood sampling, were randomly identified from the registers of the Medical Biobank of Northern Sweden. The Regional Ethics Committee at the University Hospital, Umeå, Sweden, approved this study (Dnr 2011-168-31M), and all individuals had given their written informed consent to participate in research projects.

Out of the 386 cases who later on developed RA, 88 participants had available plasma and were therefore included in the current report (Figure 1B). The pre-dating time before the diagnosis of RA was 8.5 ± 5.0 years (mean ± standard deviation, SD) [23]. The 88 cases were matched 1:1 with 88 available controls. Matched was based on sex, age, and sample year.

### 4.3. Biochemical Assessments

In the SOS study, ESR was measured at the participants’ health care centers at the time of health examination visits. CRP levels at baseline were measured with an ultrasensitive immunoturbidimetric method (Sentinel, Milan, Italy) using the Architect c8200 analyzer (Abbott Laboratories, Abbott Park, IL, USA) in Helsinki, Finland, between October 2010 and April 2011. Measurement of serum concentrations of adiponectin was performed at the German Diabetes Center, Duesseldorf, Germany, from November 2010 to April 2011 [39]. Total adiponectin was measured using the Human Total Adiponectin/Acrp30 Quantikine ELISA Kit (DRP300, Bio-Techne, Minneapolis, MN, USA, previously R&D Systems, Wiesbaden, Germany). Serum leptin, resistin, and visfatin levels were measured using Human Leptin Quantikine ELISA Kit (DLP00, Bio-Techne, Minneapolis, MN, USA), Human Resistin Quantikine ELISA Kit (DRSN00, Bio-Techne, Minneapolis, MN, USA), and Nampt (Visfatin/PBEF) human ELISA Kit (AG-45A-0006YEK-KI01, AdipoGen Life Sciences, San Diego, CA, USA) respectively, between April and June 2018 at the University of Gothenburg, Sweden. All the ELISA experiments were performed following the manufacturers’ instructions. All samples gave values above the limit of detection.

In the cohort from the Medical Biobank of Northern Sweden, plasma adiponectin, leptin, and resistin were measured using the same ELISA kits as for the SOS study. All measurements were performed at the University of Gothenburg (Sweden) in August 2018. Two samples had leptin levels above the detection range, and their values were defined as the upper detection limit. The leptin level of one sample was below the detection range, and its value was defined as 0 ng/mL. Visfatin could not be measured in the cohort from the Medical Biobank of Northern Sweden due to a lack of plasma.

### 4.4. Statistical Analysis

Data are shown as mean ± SD for continuous variables or number (percentage) for categorical variables. Spearman’s test was used to assess the correlation among adipokines. Differences between group means were analyzed using analysis of covariance, whereas proportions were analyzed using a chi-squared or Fisher’s Exact test. Multivariable conditional logistic regression analysis was used to calculate adjusted odds ratios (OR) and corresponding 95% confidence intervals (CIs) for the risk of RA in the SOS cohort and in the cohort from the Medical Biobank of Northern Sweden. Odds ratio and corresponding 95% confidence intervals for the risk of RA in the cohort from the Medical Biobank of Northern Sweden after stratifying for BMI were calculated with binary logistic regression after adjustment for preselected risk factors. All *p*-values were two-sided, and *p*-values < 0.05 were considered statistically significant. Statistical analyses were performed with the Statistical Package for Social Science (version 24.0; SPSS, Chicago, IL, USA).

## 5. Conclusions

This study shows that in subjects with overweight/obesity, increased circulating adiponectin levels were associated with a higher risk of developing RA independently of other adipokines. We were not able to detect any association between circulating levels of leptin, resistin, or visfatin and the risk of developing RA, regardless of weight.

## Figures and Tables

**Table 1:** Baseline characteristics of the nested case-control cohort from the SOS study.

Characteristic	Cases (*n* = 82)	Matched Controls (*n* = 410)	*p*-Value
Age, years *	48 ± 6	48 ± 6	0.91
Men, No (%) *	17 (21)	85 (21)	0.99
BMI, kg/m^2^ *	42 ± 5	41 ± 4	0.60
Surgery group, No (%) *	43 (52)	218 (53)	0.90
Current or previous smoking, No (%) *	18 (22)	85 (21)	0.80
Diabetes, No (%)	7 (9)	45 (11)	0.50
CRP, mg/L	10 ± 12	7 ± 7	0.001
ESR, mm/h	18 ± 11	15 ± 10	0.048
Adiponectin, ng/mL	9041 ± 4766	8068 ± 4622	0.08
Leptin, ng/mL	45 ± 32	49 ± 29	0.26
Resistin, ng/mL	12 ± 6	12 ± 6	0.84
Visfatin, ng/mL	2.8 ± 2.3	2.6 ± 1.9	0.35

Data are shown as mean ± standard deviation or numbers (percentage). Differences between group means were analyzed using analysis of covariance, whereas proportions were analyzed using a chi-squared test. Adipokines have been measured in serum samples. * Matching variables, plus year of inclusion. Abbreviations: BMI: body mass index; CRP: C-reactive protein; ESR: erythrocyte sedimentation rate.

**Table 2:** Baseline characteristics of the nested case-control cohort from the Medical Biobank of Northern Sweden.

Characteristic	Cases (*n* = 88)	Matched Controls (*n* = 88)	*p*-Value
Age, years *	56 ± 10	55 ± 10	0.54
Men, No (%) *	20 (23)	20 (23)	1.00
BMI, kg/m^2^	26 ± 4	26 ± 3	0.56
Current or previous smoking, No (%)	43 (49)	28 (32) **	0.03
Adiponectin, ng/mL	7482 ± 4936	6628 ± 4654	0.18
Leptin, ng/mL	21 ± 17	20 ± 19	0.44
Resistin, ng/mL	6.7 ± 3.5	6.9 ± 2.8	0.42

Data are shown as mean ± standard deviation or numbers (percentage). Differences between group means were analyzed using analysis of covariance, whereas proportions were analyzed using a chi-squared test. Adipokines have been measured in plasma samples. * Matching variables, plus date of blood sampling. ** Information about smoking status was missing for one individual. Abbreviations: BMI: body mass index.

**Table 3:** Multivariable conditional logistic regression analysis for RA in the nested case-control cohort from the SOS study.

**A. Model 1**	**OR**	**95% CI**	***p*-Value**
Adiponectin, per 1000 ng/mL	1.05	1.00–1.10	0.05
Leptin, per 10 ng/mL	0.93	0.84–1.03	0.16
Resistin, per 10 ng/mL	0.93	0.59–1.47	0.75
Visfatin, per 1 ng/mL	1.07	0.95–1.21	0.29
**B. Model 2**	**OR**	**95% CI**	***p*-Value**
Adiponectin, per 1000 ng/mL	1.06	1.01–1.12	0.02
Leptin, per 10 ng/mL	0.92	0.83–1.02	0.13
Resistin, per 10 ng/mL	0.67	0.40–1.15	0.15
Visfatin, per 1 ng/mL	1.04	0.91–1.19	0.57
CRP, per 10 mg/L	1.67	1.17–2.42	0.01
ESR, per 10 mm/h	1.14	0.88–1.48	0.32

Abbreviations: OR: odds ratio; CI: confidence interval; CRP: C-reactive protein; ESR: erythrocyte sedimentation rate.

**Table 4:** Multivariable conditional logistic regression analysis for RA risk (A) and logistic regression analyses after stratifying for BMI (B and C) in the Medical Biobank of Northern Sweden cohort.

Characteristic	OR	95% CI	*p*-Value
**A.**			
Adiponectin, per 1000 ng/mL	1.05	0.98–1.13	0.19
Leptin, per 10 ng/mL	1.01	0.99–1.03	0.56
Resistin, per 10 ng/mL	0.75	0.85–1.12	0.75
Smoking, yes/no	2.27	1.12–4.62	0.02
**B. BMI ≤ 25 kg/m^2^**			
Adiponectin, per 1000 ng/mL	1.02	0.94–1.11	0.64
Leptin, per 10 ng/mL	1.00	0.93–1.07	0.92
Resistin, per 10 ng/mL	1.05	0.85–1.29	0.68
Women, yes/no	1.42	0.25–8.03	0.69
Age, per 1 year	0.99	0.94–1.05	0.79
Smoking, yes/no	2.04	0.72–5.81	0.18
**C. BMI > 25 kg/m^2^**			
Adiponectin, per 1000 ng/mL	1.17	1.01–1.36	0.03
Leptin, per 10 ng/mL	1.00	0.98–1.03	0.94
Resistin, per 10 ng/mL	0.98	0.87–1.09	0.69
Women, yes/no	0.84	0.25–2.78	0.77
Age, per 1 year	1.00	0.97–1.05	0.70
Smoking, yes/no	1.63	0.71–3.72	0.25

Abbreviations: OR: odds ratio; CI: confidence interval; BMI: body mass index.

**Table 5:** Baseline characteristics of the cohort from the Medical Biobank of Northern Sweden stratified by BMI.

Characteristic	Cases	Matched Controls	*p*-Value
**BMI ≤ 25**	***n* = 30**	***n* = 37**	
Age, years	53 ± 11	54 ± 9	0.67
Women, No (%)	26 (87)	32 (87)	0.64
BMI, Kg/m^2^	22 ± 2	23 ± 1	0.13
Current or previous smoking, No (%)	15 (50)	12 (32)	0.15
Adiponectin, ng/mL	9233 ± 6575	8708 ± 5921	0.73
Leptin, ng/mL	13 ± 8	12 ± 9	0.79
Resistin, ng/mL	6.9 ± 2.5	6.8 ± 2.5	0.99
**BMI > 25**	***n* = 58**	***n* = 51**	
Age, years	57 ± 9	57 ± 10	0.63
Women, No (%)	42 (72)	36 (71)	0.83
BMI, Kg/m^2^	28 ± 3	28 ± 3	0.76
Current or previous smoking, No (%)	28 (48)	16 (31) *	0.09
Adiponectin, ng/mL	6575 ± 3570	5118 ± 2630	0.02
Leptin, ng/mL	25 ± 18	26 ± 22	0.80
Resistin, ng/mL	6.7 ± 3.9	6.9 ± 2.5	0.74

Data are shown as mean ± standard deviation or numbers (percentage). Differences between group means were analyzed using analysis of covariance, whereas proportions were analyzed using a Chi-square test or Fisher’s Exact test. * Information about smoking status was missing for one individual. Abbreviation: BMI: body mass index.

## Data Availability

All relevant data are within the manuscript and its supporting information files.
